# RETRACTION: Studying the Effects of Stress, Mental Health and Psychological Well‐Being on Wound Healing Rates After Oesophageal Varices Ligation in Liver Cirrhosis Patients

**DOI:** 10.1111/iwj.70490

**Published:** 2025-04-02

## Abstract

**RETRACTION:** YuH. X. , FuL. X. , WangY. Y. , and ZhengS. L. , “Studying the Effects of Stress, Mental Health and Psychological Well‐Being on Wound Healing Rates After Oesophageal Varices Ligation in Liver Cirrhosis Patients,” International Wound Journal 21, no. 2 (2024): e14703, 10.1111/iwj.14703.PMC1083191042052871

The above article, published online on 01 February 2024, in Wiley Online Library (http://onlinelibrary.wiley.com/), has been retracted by agreement between the journal Editor in Chief, Professor Keith Harding; and John Wiley & Sons Ltd. Following an investigation by the publisher, all parties have concluded that this article was accepted solely on the basis of a compromised peer review process. The editors have therefore decided to retract the article. The authors did not respond to our notice regarding the retraction.

**RETRACTION:** H. X. Yu , L. X. Fu , Y. Y. Wang , and S. L. Zheng , “Studying the Effects of Stress, Mental Health and Psychological Well‐Being on Wound Healing Rates After Oesophageal Varices Ligation in Liver Cirrhosis Patients,” International Wound Journal 21, no. 2 (2024): e14703, 10.1111/iwj.14703.PMC1083191042052871

The above article, published online on 01 February 2024, in Wiley Online Library (http://onlinelibrary.wiley.com/), has been retracted by agreement between the journal Editor in Chief, Professor Keith Harding; and John Wiley & Sons Ltd. Following an investigation by the publisher, all parties have concluded that this article was accepted solely on the basis of a compromised peer review process. The editors have therefore decided to retract the article. The authors did not respond to our notice regarding the retraction.

